# Relevance of oxidative stress for small intestinal injuries induced by nonsteroidal anti-inflammatory drugs: A multicenter prospective study

**DOI:** 10.1097/MD.0000000000040849

**Published:** 2024-12-13

**Authors:** Yuki Baba, Seiji Kawano, Akinobu Takaki, Yoshiyasu Kono, Joichiro Horii, Sakuma Takahashi, Daisuke Kawai, Sayo Kobayashi, Hiroyuki Okada

**Affiliations:** a Department of Internal Medicine, Japanese Red Cross Himeji Hospital, Himeji, Japan; b Department of Gastroenterology and Hepatology, Okayama University Graduate School of Medicine, Dentistry and Pharmaceutical Sciences, Okayama, Japan; c Department of Gastroenterology, National Hospital Organization Fukuyama Medical Center, Fukuyama, Japan; d Department of Gastroenterology, Kagawa Prefectural Central Hospital, Kagawa, Japan; e Department of Internal Medicine, Okayama Saiseikai General Hospital, Okayama, Japan; f Department of Internal Medicine, Fukuyama City Hospital, Fukuyama, Japan.

**Keywords:** capsule endoscopy, NSAIDs, oxidative stress, small intestinal mucosal injury

## Abstract

Several reports revealed that oxidative stress was involved in the mouse model of nonsteroidal anti-inflammatory drug (NSAIDs)-induced small intestinal mucosal injuries. Thus, we aimed to investigate in the prospective clinical study, that the relevance of oxidative stress balance in small intestinal mucosal injury in NSAIDs users. We prospectively included 60 patients who had been taking NSAIDs continuously for more than 3 months and exhibited obscure gastrointestinal bleeding (number UMIN 000011775). Small intestinal mucosal injuries were assessed by capsule endoscopy (CE), and reactive oxygen metabolites (d-ROMs) levels and oxidant capacity (OXY) adsorbent test were performed to investigate the relevance of oxidative stress balance. More than half of the patients (N = 32, 53%) had small intestinal mucosal injuries by CE, and 14 patients (24%) had ulcers. The incidence of ulcers was relatively higher in nonaspirin users. Serum OXY levels were significantly lower in the mucosal injury group (*P* = .02), and d-ROM levels were significantly higher in the ulcer group (*P* < .01). In aspirin users, d-ROM and OXY levels did not differ significantly with respect to mucosal injuries or ulcers. However, in nonaspirin users, OXY level was significantly lower in the mucosal injury group (*P* = .04), and d-ROM levels were significantly higher in the ulcer group (*P* = .02). Nonaspirin NSAIDs-induced intestinal mucosal injury is associated with antioxidant systems, resulting in increased oxidative stress.

## 1. Introduction

Long-term use of nonsteroidal anti-inflammatory drugs (NSAIDs), including low-dose aspirin, can induce upper gastrointestinal (GI) complications.^[1,2]^ However, less attention has been paid to damage induced across throughout the GI tract. The prevalence of NSAIDs-induced small intestinal injury has become clear since the development of capsule endoscopy (CE) and balloon-assisted endoscopy. Using CE, Graham et al reported that small bowel injuries were noted in 71% of patients with arthritis who took nonselective NSAIDs for more than 3 months, compared to 10% in patients that took non-NSAIDs.^[3]^ A recent review highlighted that various prospective studies with healthy volunteers who were administered short-term nonselective NSAIDs treatment confirmed the high toxicity of NSAIDs on the small bowel.^[4]^

Several potential mechanisms underlying the etiology of NSAIDs-induced small intestinal injuries have been proposed. First, a decrease in endogenous prostaglandin (PG) due to inhibition of cyclooxygenase is considered important.^[5]^ This is believed to be the main cause of NSAIDs-induced small intestinal injuries. Second, NSAIDs can directly damage mitochondria in small intestinal epithelial cells. This results in the depletion of intracellular energy and production of free radicals, which further disrupt the intercellular tight junctions of the small intestinal epithelium. Consequently, the mucosal barrier weakens, and bile acid, proteolytic enzymes, intestinal bacteria, and toxins can easily enter epithelial cells, resulting in mucosal injuries.^[6]^ Based on these hypotheses, several drugs have been tested for the prevention or treatment of NSAIDs-induced small intestinal injuries. Specifically, PG analogs (namely misoprostol and rebamipide), the gap-junction intercellular communication agent irsogladine, and probiotics have been reported to be effective.^[7–10]^ However, the best approach for the treatment of NSAIDs-induced small intestinal injuries has not yet been clearly defined. Therefore, novel therapeutic approaches are urgently needed for the same. A number of areas need to be explored from mechanistic point of view, role of mitochondria and other organelles in NSAIDs-pathophysiology along with genetic polymorphisms plausibly linked with NSAIDs-susceptibility.

Previously, we focused on the mechanism of NSAIDs-induced injury to the mitochondrial pathway and the effect of L-carnitine (LC), an oxidative stress scavenger that also supports mitochondrial function. Oxidative stress was involved in the mouse model of NSAIDs-induced small intestinal mucosal injuries. Antioxidants, especially the mitochondrial supporting agent LC, were good candidates for preventing intestinal mucosal injury. We hypothesized that its effect should be different from standard antioxidants and reported the effectiveness of LC on NSAIDs-induced small intestinal mucosal injuries in a mouse model.^[11]^ The aim of the prospective clinical study reported in the present study was 2-fold. One was the evaluation of the actual state of small intestinal mucosal injuries using CE in long-term NSAIDs users. The other was the investigation of the relevance of oxidative stress balance in small intestinal mucosal injury in NSAIDs users.

## 2. Materials and methods

### 2.1. Patients

We prospectively assessed oxidative stress status of NSAID users. The study included 60 patients who between September 2013 to September 2019 had been taking NSAIDs (including aspirin) continuously for more than 3 months and exhibited obscure gastrointestinal bleeding, which is defined as an anemia and/or fecal blood-positive symptoms with no upper or lower gastrointestinal bleeding lesions detectable by endoscopy. The patients underwent CE at 5 hospitals in the Okayama Gut Study Group. The inclusion criteria were as follows: age ≥ 20 years; no upper and lower gastrointestinal injures as detected by endoscopy; and no history of gastrointestinal obstruction or stenosis. The study protocol was approved by the Okayama University Hospital and the ethical committees of each hospital involved in the Okayama Gut Study Group, and written informed consent was obtained from all study participants. This study was registered in the UMIN Clinical Trial Registry (number UMIN 000011775).

### 2.2. CE examination

CE was performed using a PillCam SB2 or SB3 (Given Imaging Ltd, Yokneam, Israel). The images were analyzed with RAPID Reader 6.5 or 8 software on a RAPID workstation (both from Given Imaging Ltd). All images were reviewed by an expert gastroenterologist at each institution. Positivity for small intestinal mucosal injuries was defined as no less than 3 mucosal breaks (i.e., erosions or ulcers) on CE.

### 2.3. Measurement of serum reactive oxygen metabolite levels and antioxidant capacity

Reactive oxygen metabolites (d-ROMs) levels in the blood were accepted as markers of circulating reactive oxygen species (ROS). Hence, serum d-ROM levels were measured using a spectrophotometer (Diacron International, Grosseto, Italy) as described previously.^[12]^ To determine total plasma antioxidant capacity, an oxidant capacity (OXY) adsorbent test was performed using a spectrophotometer (Diacron International).

### 2.4. Statistical analysis

The results are expressed as the mean ± standard deviation for parametric data and medians for nonparametric data. Parametric data were compared using the Student *t* test, Tukey test, and Dunnett test. Nonparametric data were compared using the Mann–Whitney *U*-test, Steel–Dwass test, and Steel test. Categorical data were analyzed using Fisher exact test or the chi-squared test. Data were considered statistically significant at *P* < .05.

## 3. Results

### 3.1. Clinical parameters in all subjects

The clinical characteristics of the patients are presented in Table 1. Among the 60 patients enrolled in this study, 28 (47%) were male, and the median age was 77 years. Patients were divided into 3 groups based on the use of NSAIDs: aspirin users (N = 28), nonaspirin users (N = 28), and combined aspirin and nonaspirin users (N = 4). Among the comorbidities, hypertension was the most common (29/60, 49%), followed by ischemic heart disease (24/60,41%), chronic kidney disease (CKD) (15/60, 25%), orthopedic disease or rheumatic disease, and diabetes mellitus (20%). Among the concomitant medications, 34 patients (58%) were taking proton pump inhibitors (PPI), 15 patients (26%) were taking anticoagulant drugs, and 8 patients were taking nonaspirin antiplatelet drugs (14%). More than half of the patients (N = 32, 53%) had small intestinal mucosal injuries (≥3 mucosal breaks) as assessed by CE, and 14 patients (24%) had ulcers.

**Table 1:** Patient’s characteristics in the enrolled study.

Patient’s characteristics	N = 60
Sex: male/female	28/32
Median age (yr)	77
NSAIDs	
Aspirin/nonaspirin/combined	28/28/4
Comorbidity, n (%)	
Hypertension	29 (49)
Ischemic heart disease	24 (41)
Chronic kidney disease	15 (25)
Orthopedic disease or rheumatic disease	13 (22)
Diabetes mellitus	12 (20)
Liver cirrhosis	6 (10)
Cerebrovascular disease	5 (8)
Concomitant medication	
PPI, n (%)	34 (58)
H_2_RA, n (%)	9 (15)
Gastroprotective agent	20 (34)
Anticoagulant drugs, n (%)	15 (25)
Antiplatelet drugs (nonaspirin)	8 (25)
Mucosal injury, n (%)	32 (58)
Ulcer, n (%)	14 (23)
Erosion, n (%)	18 (35)

### 3.2. Comparison of clinical parameters between mucosal injury/ulcer positive group and negative group

Tables 2 and 3 show the clinical characteristics of the mucosal injury group versus the noninjury group, and the ulcer group versus the non-ulcer group, respectively. The detection rate of total mucosal injury was not significantly different between the groups based on the types of NSAIDs; however, the incidence of mucosal ulcers was relatively higher in nonaspirin users (*P* = .04). No comorbidities, such as ischemic heart disease and CKD, were significantly different between the groups.

**Table 2:** Clinical characteristics of mucosal injury group compared with noninjury group.

	Mucosal injury group (n = 32)	Noninjury group (n = 28)	
Age (yr)	73 ± 11	72 ± 18	NS
Sex (n, male/female)	11/21	18/10	0.04
Aspirin/nonaspirin/combined	12/17/3	18/13/1	NS
Ischemic heart disease	16	8	NS
Diabetes	9	6	NS
OGIB type occult/overt	12/20	13/15	NS
Rebleeding	5	4	NS
Indication of therapy	26	11	<0.001

NS = not significant.

**Table 3:** Clinical characteristics of ulcer group compared with non-ulcer group.

	Ulcer group (n = 14)	Non-ulcer group (n = 46)	
Age (yr)	72 ± 14	73 ± 14	NS
Sex (n, male/female)	6/8	23/23	NS
Aspirin/nonaspirin/combined	3/8/3	28/17/1	0.04
Ischemic heart disease	8	16	NS
Chronic kidney disease	4	11	NS
OGIB type occult/overt	4/10	21/25	NS
Rebleeding	3	6	NS
Indication of therapy	14	23	<0.001

NS = not significant.

### 3.3. Characteristics in the analysis of serum oxidative stress

Serum levels of d-ROMs and antioxidant capacity were assessed in 53 patients. Patients who were took a combination of aspirin and nonaspirin (N = 4) were excluded from the analysis. 3 patients were poor condition of serum preservation. There were no significant differences between the aspirin and nonaspirin groups in terms of age or sex. The percentage of patients who were on anticoagulants (*P* = .02) and had ischemic heart disease or CKD (*P* < .01) was significantly higher in the aspirin group than that in the nonaspirin group (Table 4).

**Table 4:** Patient’s characteristics in the analysis of serum oxidative stress.

	Total (n = 53)	Aspirin (n = 26)	Nonaspirin (n = 27)	*P* value
Age (yr, median)	76	77	75	NS
Sex (n, M/F)	28/25	13/13	15/12	NS
Concomitant medication (n, %)				
PPI	31 (58)	18 (69)	13 (48)	NS
H2-RA	6 (11)	3 (12)	3 (11)	NS
Gastroprotective agent	18 (34)	7 (27)	11 (41)	NS
Antiplatelet drugs (nonaspirin)	7 (13)	4 (15)	3 (11)	NS
Anticoagulant drugs	13 (25)	10 (38)	3 (11)	.018
Comorbidity (n, %)				
Hypertension	25 (47)	14 (54)	11 (41)	NS
Ischemic heart disease	20 (38)	18 (69)	2 (7)	<.01
Chronic kidney disease	12 (23)	10 (38)	2 (7)	<.01
Orthopedic disease or Rheumatic disease	12 (23)	5 (19)	7 (26)	NS
Diabetes mellitus	12 (23)	9 (35)	3 (11)	.038
Liver cirrhosis	6 (11)	3 (12)	3 (11)	NS
Cerebrovascular disease	4 (8)	2 (8)	2 (7)	NS

NS = not significant.

### 3.4. Comparison of serum d-ROM and OXY levels

**Figure 1.**
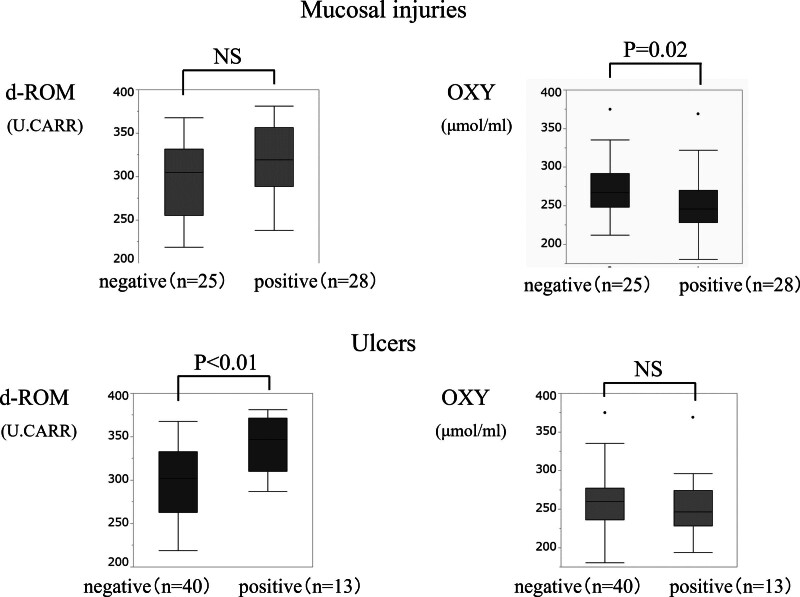
Comparison of serum d-ROM and OXY levels in the study population. Median serum OXY levels were significantly lower in the mucosal injury group compared to that in the non-mucosal injury group (*P* = .02). d-ROM levels were significantly higher in the ulcer group compared to that in the non-ulcer group (*P* < .01). d-ROM = reactive oxygen metabolite. OXY = oxidant capacity.

As shown in Figure 1, of the 53 patients, 32 patients (60%) had small intestinal mucosal injuries, while 13 patients (25%) had ulcers. Median serum OXY levels were significantly lower in the mucosal injury group compared to that in the non-mucosal injury group (*P* = .02). In contrast, d-ROM levels were significantly higher in the ulcer group compared to that in the non-ulcer group (*P* < .01).

### 3.5. Comparison of serum d-ROM and OXY levels in aspirin or nonaspirin users

**Figure 2.**
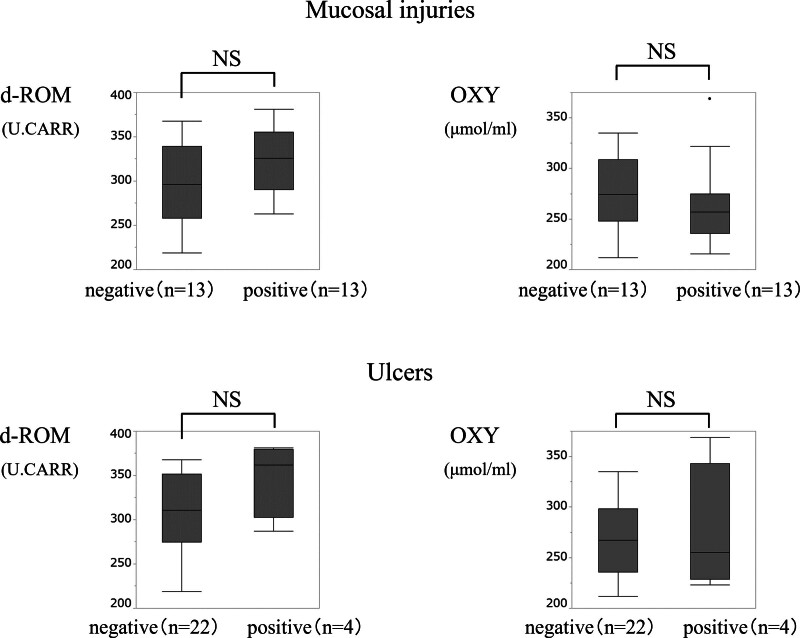
Comparison of serum d-ROM and OXY levels in aspirin users. d-ROM and OXY levels did not differ significantly with respect to mucosal injuries or ulcers. d-ROM = reactive oxygen metabolite. OXY = oxidant capacity.

**Figure 3.**
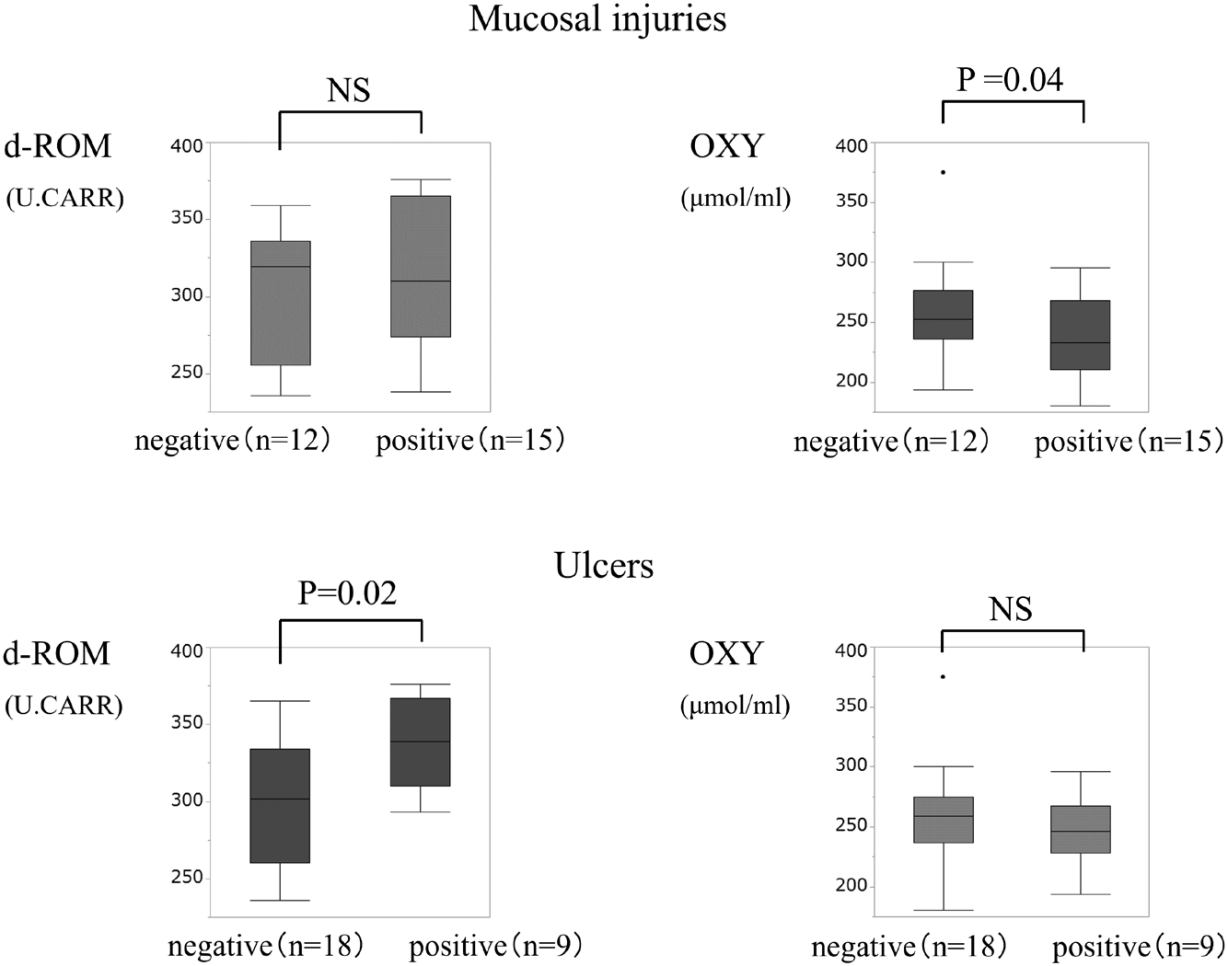
Comparison of d-ROM and OXY levels in nonaspirin users. Median serum OXY level was significantly lower in the mucosal injury group compared to that in the non-mucosal injury group (*P* = .04), and d-ROM levels were significantly higher in the ulcer group compared to that in the non-ulcer group (*P* = .02). d-ROM = reactive oxygen metabolite, OXY = oxidant capacity.

For additional analysis, we categorized the patients into aspirin and nonaspirin user s. Serum d-ROM and OXY levels in each group are shown in Figures 2 and 3. In aspirin users, d-ROM and OXY levels did not differ significantly with respect to mucosal injuries or ulcers. However, in nonaspirin users, the median serum OXY level was significantly lower in the mucosal injury group compared to that in the non-mucosal injury group (*P* = .04), and d-ROM levels were significantly higher in the ulcer group compared to that in the non-ulcer group (*P* = .02).

## 4. Discussion

To the best of our knowledge, this prospective study is the first to investigate the relationship between oxidative stress and NSAIDs-induced small intestinal mucosal injuries. The median serum OXY level was significantly lower in patients with no fewer than 3 mucosal breaks, while the d-ROM level was significantly higher in patients with ulcers. Interestingly, these variations in oxidative stress were remarkable in the nonaspirin group.

PG deficiency is the major factor underlying NSAIDs-induced upper GI and small intestinal injuries. However, different pathophysiological mechanisms exist for these injuries, and the risk factors for NSAIDs-induced small intestinal injuries have not yet been completely established. In previous studies, the molecular mechanisms underlying NSAIDs-induced intestinal injuries were classified into PG-dependent and PG-independent mechanisms.^[13]^

The contribution of the gut microbiome to NSAIDs-induced enteropathy is an important PG-independent factor. Both laboratory and clinical studies have demonstrated that PPI use can potentially aggravate NSAIDs-induced small intestinal injuries.^[14,15]^ Furthermore, animal studies strongly suggest that enterobacteria, especially Gram-negative bacteria, are the most important factors for intestinal ulceration induced by NSAIDs.^[16]^

Mitochondrial dysfunction is another important factor involved in NSAID-induced small intestine injuries. Many NSAIDs directly cause mitochondrial dysfunction by uncoupling oxidative phosphorylation induced by the opening of the mega channel in the mitochondrial membrane, which is the mitochondrial permeability transition pore. This can induce the production of ROS, disturbance of mucosal barrier function, and elevation of small intestinal permeability, which play important roles in the early processes underlying NSAIDs-induced small bowel injuries.^[17]^

In the present prospective study, oxidative stress-related serum d-ROMs and OXY levels did not differ significantly between the 2 groups regardless of mucosal injury in aspirin users. Therefore, it is necessary to distinguish between nonaspirin and aspirin use when considering the effects of oxidative stress on NSAIDs-induced small intestinal mucosal injuries. Nonaspirin users with small intestinal injuries have been reported to have defects in antioxidant reservoirs.^[17]^ In contrast, aspirin users predominantly had comorbidities such as ischemic heart disease, CKD, and diabetes mellitus, as shown in Table 1. These diseases are known to be strong and multiple interconnections between oxidative stress.^[18,19]^ Therefore, the general oxidative stress balance defect probably overcomes the intestinal injury. We believe that this is the reason why no significant differences were observed in either d-ROMs or OXY in the aspirin group. On the other hand, the serum OXY levels were lower in small intestinal mucosal injuries, and d-ROMs were higher in ulcers among nonaspirin NSAIDs users. Therefore, this study suggests that further examination of relationship between NSAIDs and oxidative stress should be performed only in nonaspirin NSAID cases.

ROS are produced continuously during normal physiological events and can easily initiate the peroxidation of membrane lipids, leading to the accumulation of lipid peroxides. However, these compounds are cleared via antioxidant defense mechanisms. Hence, there is a balance between ROS generation and ROS inactivation by antioxidant systems in organisms. Under pathological conditions, there is overproduction of ROS, resulting in oxidative stress. ROS are generated when endogenous antioxidant defenses are inadequate. An imbalance between ROS production and antioxidant defense mechanisms leads to the oxidative modification of cellular membranes or intracellular molecules.^[20]^ In the present study, we confirmed that NSAIDs-induced mucosal injuries were related to oxidative stress and imbalances in the antioxidative systems. Therefore, antioxidant drugs can potentially be used for the prevention of NSAIDs-induced small intestinal injuries.

We have previously reported the effectiveness of LC in NSAIDs-induced small intestinal mucosal injuries in a mouse model.^[11]^ In that manuscript, we have already conducted a pilot research and reported the small amount of clinical data. These cases were also included in the cases in this study. However, the increase of the number of cases in this study enabled us to conduct a more detailed analysis based on actual clinical practice, such as by separating ulcer from mucosal injury, and by separating aspirin from nonaspirin NSAIDs. Recently, other studies have reported the therapeutic potential of uric acid and hydrogen-rich water for small intestinal inflammatory diseases.^[21,22]^ However, there have been no clinical reports on the effectiveness of antioxidant drugs for small intestinal injuries. The results of this clinical study may provide new possibilities for the treatment of NSAIDs-induced small intestinal injuries.

This study has several limitations. First, number of patients participated in the study was relatively small. Second, analysis of CE images and diagnosis were performed by the endoscopists in charge of each facility. However, in all the facilities in this study specialists were in charge of the interpretation. Finally, the condition of serum storage might be different because of the multicenter study, however, measurement of markers with oxidative stress were performed by the same inspector under the same conditions.

In conclusion, nonaspirin NSAIDs-induced intestinal mucosal injury is associated with decreased/defective antioxidant systems, resulting in increased oxidative stress. Hence, antioxidants can potentially be used to prevent nonaspirin NSAIDs-induced intestinal mucosal injury.

## Author contributions

**Conceptualization:** Seiji Kawano, Akinobu Takaki.

**Data curation:** Yuki Baba, Seiji Kawano, Yoshiyasu Kono, Joichiro Horii, Sakuma Takahashi, Daisuke Kawai, Sayo Kobayashi.

**Formal analysis:** Seiji Kawano.

**Investigation:** Akinobu Takaki.

**Supervision:** Akinobu Takaki, Hiroyuki Okada.

**Validation:** Akinobu Takaki.

**Writing – original draft:** Yuki Baba.

**Writing – review & editing:** Seiji Kawano.
